# Depletion of skeletal muscle mass adversely affects long-term outcomes for men undergoing gastrectomy for gastric cancer

**DOI:** 10.1371/journal.pone.0256365

**Published:** 2021-08-19

**Authors:** Katsunobu Sakurai, Naoshi Kubo, Yutaka Tamamori, Naoki Aomatsu, Takafumi Nishii, Akiko Tachimori, Yukio Nishiguchi, Kiyoshi Maeda

**Affiliations:** 1 Department of Gastroenterological Surgery, Osaka City General Hospital, Osaka, Japan; 2 Department of Gastroenterological Surgery, Osaka City Juso Hospital, Osaka, Japan; Ehime University Graduate School of Medicine, JAPAN

## Abstract

**Background:**

Although low skeletal muscle mass has an adverse impact on the treatment outcomes of cancer patients, whether the relationship between preoperative skeletal muscle mass and gastrectomy outcomes in gastric cancer (GC) differs between men and women is unclear. The study aimed to clarify this relationship based on gender.

**Methods:**

Between January 2007 and December 2015, 1054 patients who underwent gastrectomy for GC at Osaka City General Hospital were enrolled in this study. We evaluated sarcopenia by the skeletal muscle index (SMI), which was measured by computed tomography (CT) using areas of muscle in the third lumbar vertebral body (L3). Male and female patients were each divided into two groups (low skeletal muscle and high skeletal muscle).

**Results:**

The SMI emerged as an independent predictor of 5-year overall survival (OS) in male GC patients (Hazard ratio 2.51; 95% confidence interval (CI) 1.73–3.63, *p* < 0.001) based on multivariate analysis. However, this index was not an independent predictive determinant of 5-year cancer-specific survival (CSS). The SMI was not an independent predictor of either OS or CSS in female GC patients. The incidence of leakage and major complication (Clavien Dindo grade ≧ 3) did not differ significantly across groups.

**Conclusions:**

Preoperative skeletal muscle mass is a valuable prognostic predictor of OS in male GC patients.

## Introduction

In general, women live longer than men. The long-term outcomes for cancer patients are also better for women than men based on large studies conducted in European countries [1, 2]. Although these studies suggested that biological factors, such as hormonal status, may affect a woman’s longevity, the mechanism of the female survival advantage has not yet been clarified.

Recently, sarcopenia (i.e., loss of skeletal muscle mass) was reported as a paramount nutritional parameter predictive of therapeutic outcomes for various diseases, including cancer [3]. Typically, skeletal muscle mass is greater in men than in women. Although the effects of muscle on the body are expected to differ between genders, the evaluation of sarcopenia in cancer patients by gender has only been performed in one previous study [4], in which they found that sarcopenia only had an adverse impact on the outcomes of male lymphoma patients.

For gastric cancer (GC), preoperative malnutrition (e.g., low body mass index [BMI] or prognostic nutritional index [PNI]) worsens treatment outcomes [5, 6]. Thus, nutritional assessment before gastrectomy is crucial. Several reports showed that preoperative skeletal muscle mass could predict gastrectomy outcomes for GC patients [7, 8]. However, there have been no reports on gender differences for the impact of muscle mass on treatment outcomes of GC patients following gastrectomy. In this study, we investigated the skeletal muscle mass impact on gastrectomy outcomes of GC patients based on gender.

## Methods

### Patients and data collection

A total of 1054 patients that underwent gastrectomy for GC at the Department of Gastroenterological Surgery, Osaka city general hospital between January 2007 and December 2015 were reviewed for this study. Patients who had prior gastrectomy or did not undergo preoperative CT examinations at our hospital were excluded. We defined areas of skeletal muscle at the level of the third lumbar vertebral body (L3) in CT images and normalized them by height in meters squared (m^2^) to generate the lumbar skeletal muscle index (SMI, cm^2^/m^2^). Male and female patients were each divided into low- and the high SMI groups. We adopted gender specific cut-off values for the SMI that previous studies in GC patients have established (40.8 cm^2^/m^2^ for men and 34.9cm^2^/m^2^ for women) [8–12].

The clinicopathologic data included information on demographics, tumor characteristics, operative details, and survival. Data on perioperative morbidity and mortality were extracted from medical records. All pathologic terms and classifications were stipulated by the Japanese Classification of Gastric Carcinoma, 3^rd^ English edition [13]. Any complications were scored using the Clavien-Dindo (CD) classification, with major complications being defined as CD grade ≥ 3 [14]. Informed consent was obtained in the form of opt-out on the hospital’s web-site because the analysis used anonymous clinical data that were obtained after each patient agreed to treatment by written consent. The opt-out is a method of publishing information on the web without directly obtaining patient consent for clinical research, and ensuring that patients have the opportunity to refuse. This study was approved by the Institutional Review Board at Osaka city general hospital (IRB number: 2003141). The IRB of our hospital approved the use of an opt-out consent mechanism in this study.

### Evaluation of sarcopenia by CT images

To assess sarcopenia, we reviewed the initial CT images that were conducted for clinical staging of GC as previously described [7]. The lumbar vertebrae and adjacent muscles, including the rectus abdominus, abdominal laterals and obliques, and psoas and paraspinal muscles (quadratus lumborum, erector spinae) were evaluated. Using the volume analyzer SYNAPSE VINCENT (Fujifilm, Tokyo, Japan), tissues were differentially colorized in CT images based on Hounsfield units (HUs). A range of -29 to 150 HUs was selected to quantify muscle, as previously described [3]. The corresponding areas were displayed in green to distinguish them from the surrounding tissues. Cross-sectional areas were automatically calculated and then divided by the square of the height to generate the SMI (cm^2^/m^2^).

### Statistical analysis

Comparisons of the categorical variables between the low and high SMI groups were performed using the Pearson’s χ^2^ or Fisher exact tests. Comparisons of the continuous variables were performed using the Mann-Whitney U test. Overall survival (OS) was defined as the time from surgery until death, and cancer-specific survival (CSS) was defined as the time from surgery until death from GC. The survival curves were generated using the Kaplan-Meier method, and differences were determined by the log-rank test. Univariate and multivariate hazard ratios were calculated using the Cox proportional hazard model, and all significant variables in the univariate analysis were subjected to multivariate analysis. All analyzed *p*-values were two-sided, with *p* < 0.05 considered statistically significant. All analyses were performed using JMP v. 10 software (SAS Institute Inc, Cary, NC, USA).

## Results

### Clinical characteristics

**Fig 1:**
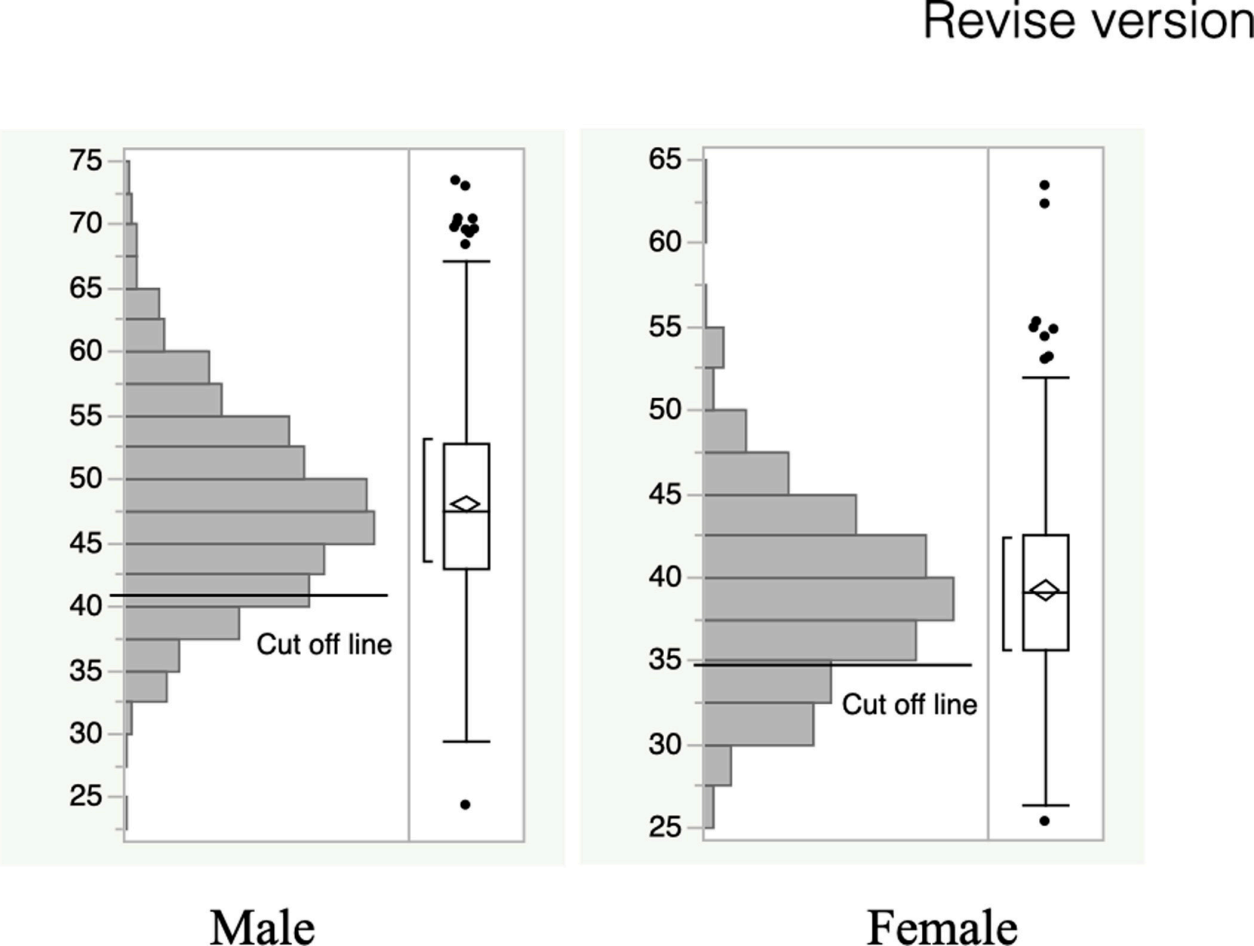
Distribution of skeletal muscle index (SMI) in men and women.

The SMI distribution is shown in Fig 1. In men, the values ranged from 24.4 to 73.4 (mean 48.1, median 47.6, first quartile 42.9, third quartile 52.8). The range was 25.4 to 63.4 in women (mean 39.3, median 39.2, first quartile 35.7, third quartile 42.5). Both had normal distributions. Male and female patients were further assigned to either the low- or high-SMI groups using gender-specific cut-off values that were 34.9 cm^2^/m^2^ for women and 40.8 cm^2^/m^2^ for men [8–12].

The clinicopathologic and perioperative characteristics of the two SMI groups of the GC patients based on gender are shown in Table 1. For men, the mean age was significantly higher in the low-SMI group compared to the high-SMI group (*p* < 0.001). The BMI was significantly lower in the low-SMI group than the high-SMI group regardless of gender (*p* < 0.001 for both). Moreover, the tumor depth was more developed in the low-SMI group compared to the high-SMI for both male and female patients (*p* = 0.015 and 0.055, respectively). Pathologic staging was significantly greater in the low-SMI group in men only (*p* = 0.012).

**Table 1:** Clinicopathologic characteristics in the low- and high-SMI groups of men and women patients.

	Male group			Female group		
	Low SMI	High SMI	*P*	Low SMI	High SMI	*P*
	N = 117	N = 574		N = 76	N = 287	
Age (yr)	71.8±10.5	65.6±10.9	<0.001	63.0±12.9	65.0±13.5	0.109
BMI (kg/m^2^)	20.1±2.5	23.7±3.1	<0.001	19.3±2.4	22.7±3.7	<0.001
Depth of tumor (pT) 1	44(37.6)	299(52.1)	0.015	33(43.4)	173(60.3)	0.055
2	17(14.5)	82(14.3)		13(17.1)	28(9.8)	
3	36(30.8)	112(19.5)		14(18.4)	41(14.3)	
4	20(17.1)	81(14.1)		16(21.1)	45(15.7)	
Lymph node metastasis (pN) 0	67(57.3)	375(65.3)	0.359	53(69.7)	197(68.6)	0.891
1	23(19.7)	90(15.7)		8(10.5)	39(13.6)	
2	13(11.1)	60(10.5)		6(7.9)	22(7.7)	
3	14(12.0)	49(8.5)		9(11.8)	29(10.1)	
pStage 1	51(43.6)	336(58.5)	0.012	40(52.6)	180(62.7)	0.275
2	38(32.5)	133(23.2)		20(26.3)	58(20.2)	
3	28(23.9)	105(18.3)		16(21.1)	49(17.1)	
Gastrectomy Total	28(23.9)	174(30.3)	0.167	14(18.4)	52(18.1)	0.952
Partial	89(76.1)	400(69.7)		62(81.6)	235(81.9)	
Intraoperative blood loss (ml)	203±251	192±239	0.720	192±476	119±195	0.365
Complication Leak	9(7.7)	30(5.2)	0.292	0	7(2.4)	0.169
Major complication	18(15.4)	59(10.3)	0.110	3(4.0)	16(5.6)	0.571
Postoperative days	19.7±16.2	17.6±13.8	0.006	14.4±10.0	15.2±9.3	0.174
Postoperative mortality 30 days	0 (0)	1 (0.2)	0.651	1 (1.3)	1 (0.4)	0.311

Values are mean (standard deviation) or number (%).

BMI, body mass index; pT, pathological T stage; pN, pathological N stage; pStage, pathological stage; Major complication indicates Clavien-Dindo grade 3 and more.

The type of gastrectomy procedures did not differ significantly by group for both male and female patients. Intraoperative blood loss did not differ significantly between either SMI group regardless of gender. There were no significant differences in leakage and major complications (≥ Grade 3) between the two SMI groups for both men and women. Postoperative stays were significantly longer in the low-SMI group in men, but this difference did not reach statistical significance (*p* = 0.006). Postoperative mortality did not differ significantly in 30 days between the low- and high-SMI group regardless of gender.

### Impact of SMI on survival

**Fig 2:**
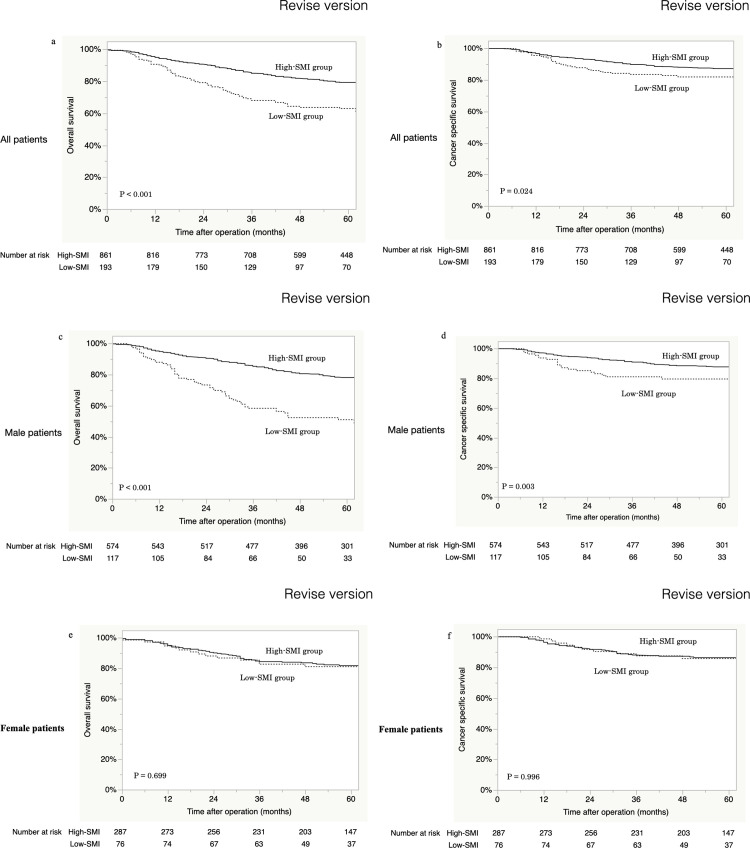
Overall (a) and cancer-specific (b) survival according to SMI status in all patients. Overall (c) and cancer-specific (d) survival according to SMI status in male patients. Overall survival (e) and cancer-specific survival (f) according to SMI status in female patients.

For the entire cohort of patients, the median follow-up was 59 months (range, 0–127 months). The 5-year OS rates for low- and high-SMI patients were 62.9% and 79.3%, respectively (*p* < 0.001, Fig 2A). The 5-year CSS rates for low- and high-SMI patients were 82.0% and 87.3%, respectively (*p* = 0.024, Fig 2B). Patients with a low SMI had significantly worse OS and CSS rates than patients with a high SMI. For men, the 5-year OS rates for low- and high-SMI patients were 50.9% and 78.2%, respectively (*p* < 0.001, Fig 2C). The 5-year CSS rates for the low- and high-SMI patients were 79.6% and 87.7%, respectively (*p* = 0.003, Fig 2D). Male patients with a low SMI had significantly worse OS and CSS rates than those with a high SMI. In contrast, the 5-year OS rates for low- and high-SMI female patients were 81.1% and 81.8%, respectively (*p* = 0.699, Fig 2E), and the 5-year CSS rates for low- and high-SMI female patients were 85.8% and 86.4%, respectively (*p* = 0.996, Fig 2F). For the female group, the patients with a low-SMI had statistically similar long-term outcomes as patients with high-SMI.

### Predictive factors for OS and CSS

Univariate analysis of OS in all patients indicated that age, gender, BMI, depth of tumor, lymph node metastasis, total gastrectomy, major complications, and SMI were independent predictors. Based on multivariate analysis, age, depth of tumor, lymph node metastasis, and SMI were independently associated with unfavorable outcomes. The hazard ratio for low SMI was 1.96 (95% CI 1.42–2.68, *p* < 0.001) (Table 2). For male GC patients, multivariate analysis indicated that SMI was an independent predictive factor for OS. The hazard ratio for low SMI was 2.51 (95% CI 1.73–3.63, *p* < 0.001) (Table 2).

**Table 2:** Univariate and multivariate analysis of overall survival.

	All patients				Male patients			
	Univariate analysis		Multivariate analysis		Univariate analysis		Multivariate analysis	
	HR (95%CI)	*P*	HR (95%CI)	*P*	HR (95%CI)	*P*	HR (95%CI)	*P*
Age (67≦)	2.39 (1.84–3.13)	<0.001	2.21 (1.69–2.91)	<0.001	2.49 (1.82–3.45)	<0.001	2.24 (1.63–3.12)	<0.001
Gender (male)	1.48 (1.13–1.96)	0.004	1.30 (0.98–1.75)	0.064	-	-		
BMI (<22)	1.39 (1.08–1.78)	0.009	1.03 (0.77–1.37)	0.858	1.67 (1.25–2.24)	0.001	1.06 (0.75–1.49)	0.745
Tumor depth (≧pT3)	3.83 (2.98–4.95)	<0.001	2.20 (1.64–2.97)	<0.001	3.14 (2.34–4.21)	<0.001	1.96 (1.39–2.77)	<0.001
Lymph node metastasis	3.85 (2.99–4.98)	<0.001	2.41 (1.81–3.23)	<0.001	3.16 (2.36–4.25)	<0.001	2.11 (1.52–2.96)	<0.001
Gastrectomy (total)	1.75 (1.35–2.26)	<0.001	1.14 (0.86–1.50)	0.354	1.51 (1.11–2.03)	0.008	1.12 (0.81–1.55)	0.481
Major complication	2.19 (1.54–3.05)	<0.001	1.36 (0.94–1.93)	0.097	2.00 (1.35–2.88)	0.001	1.32 (0.88–1.93)	0.177
SMI (low)	2.18 (1.65–2.84)	<0.001	1.96 (1.42–2.68)	<0.001	3.04 (2.21–4.13)	<0.001	2.51 (1.73–3.63)	<0.001

BMI, body mass index; pT, pathological T stage; Major complication indicates Clavien-Dindo grade 3 and more; SMI, skeletal muscle index.

Univariate analysis of CSS for all patients indicated that SMI was a predictive factor, but was not an independent predictive factor following multivariate analysis (univariate HR 1.57, 95% CI 1.04–2.31, *p* = 0.032; multivariate HR 1.37, 95% CI 0.90–2.02, *p* = 0.140). For the analysis of CSS, the SMI was a predictive factor for the CSS of male patients by univariable analysis; however, it was not an independent predictive factor based on multivariate analysis (univariate HR 2.05, 95% CI 1.23–3.27, *p* = 0.007; multivariate HR 1.67; 95% CI 0.99–2.70, *p* = 0.053) (Table 3). For women, the SMI was not a predictive factor for either OS or CSS (Table 4).

**Table 3:** Univariate and multivariate analysis of cancer-specific survival.

	All patients				Male patients			
	Univariate analysis		Multivariate analysis		Univariate analysis		Multivariate analysis	
	HR (95%CI)	*P*	HR (95%CI)	*P*	HR (95%CI)	*P*	HR (95%CI)	*P*
Age (67≦)	1.33 (0.95–1.87)	0.097	-	-	1.25 (0.82–1.92)	0.306	-	-
Gender (male)	0.95 (0.67–1.36)	0.787	-	-	-	-	-	-
BMI (<22)	1.32 (0.95–1.86)	0.102	-	-	1.33 (0.87–2.03)	0.189	-	-
Tumor depth (≧pT3)	16.45 (10.18–28.36)	<0.001	7.46 (4.39–13.42)	<0.001	21.38 (10.99–48.08)	<0.001	10.15 (4.91–23.85)	<0.001
Lymph node metastasis	11.43 (7.44–18.36)	<0.001	4.47 (2.81–7.43)	<0.001	11.14 (6.49–20.60)	<0.001	3.97 (2.22–7.60)	<0.001
Gastrectomy (total)	2.57 (1.83–3.60)	<0.001	1.22 (0.86–1.74)	0.263	2.40 (1.57–3.65)	<0.001	1.16 (0.75–1.81)	0.502
Major complication	1.84 (1.08–2.94)	0.025	1.06 (0.62–1.71)	0.828	1.94 (1.07–3.28)	0.031	1.11 (0.61–1.91)	0.718
SMI (low)	1.57 (1.04–2.31)	0.032	1.37 (0.90–2.02)	0.140	2.05 (1.23–3.27)	0.007	1.67 (0.99–2.70)	0.053

BMI, body mass index; pT, pathological T stage; Major complication indicates Clavien-Dindo grade 3 and more; SMI, skeletal muscle index.

**Table 4:** Univariate and multivariate analysis of overall survival and cancer-specific survival in women.

	Overall survival				Cancer-specific survival			
	Univariate analysis		Multivariate analysis		Univariate analysis		Multivariate analysis	
	HR (95%CI)	*P*	HR (95%CI)	*P*	HR (95%CI)	*P*	HR (95%CI)	*P*
Age (67≦)	1.99 (1.22–3.31)	0.005	1.90 (1.16–3.19)	0.011	1.45 (0.83–2.58)	0.192	-	-
BMI (<22)	1.08 (0.66–1.76)	0.769	-	-	1.30 (0.74–2.35)	0.365	-	-
Tumor depth (≧pT3)	6.41 (3.85–11.1)	<0.001	3.25 (1.81–6.04)	<0.001	12.0 (6.07–26.36)	<0.001	5.00 (2.35–11.77)	<0.001
Lymph node metastasis	6.40 (3.85–11.07)	<0.001	3.51 (1.98–6.44)	<0.001	12.0 (6.08–26.38)	<0.001	5.54 (2.64–12.86)	<0.001
Gastrectomy (total)	2.36 (1.39–3.89)	0.002	1.35 (0.78–2.27)	0.274	3.04 (1.68–5.38)	<0.001	1.51 (0.82–2.71)	0.186
Major complication	2.36 (0.91–5.02)	0.074	-	-	1.58 (0.38–4.33)	0.471	-	-
SMI (low)	1.12 (0.61–1.94)	0.703	-	-	1.00 (0.47–1.93)	0.996	-	-

BMI, body mass index; pT, pathological T stage; Major complication indicates Clavien-Dindo grade 3 and more; SMI, skeletal muscle index

## Discussion

In this study, we sought to identify gender differences in the short- and long-term outcomes of sarcopenic GC patients by using CT-measured skeletal muscle mass. We found that the SMI was independently predictive of OS in male GC patients. In contrast, no significant differences were observed between the OS of low- and high-SMI female GC patients. This study is the first to report that the impact of muscle mass on OS varies in GC patients based on gender. In CSS, male, but not female, patients with a low SMI had significantly worse prognosis. However, SMI was not an independent predictor of CSS in male patients. We think that the results may be related to the sample size of male patients. A larger multicenter study is needed to valid our results.

This finding suggests that SMI may have greater potential to predict the prognosis of male GC patients compared to female GC patients. In our view, patients with a low SMI may have greater malnutrition preoperatively, lower immunity postoperatively, and a higher risk of death from other diseases, even if GC was removed. Klein et al. reported that males and females have robust differences in their susceptibility to various autoimmune and infectious diseases, emphasizing that sex is an important biological variable that affects the immune system [15]. Thus, gastrectomy for low-SMI male GC patients may have a greater negative impact on the immune system than it would on the immune system of low-SMI female patients. Our data suggest that male patients with low SMI should be given intensive nutritional guidance to improve skeletal muscle mass before and after surgery. Whether such strategies could enhance outcomes in GC patients remains unclear. More work is needed to address these issues.

The mechanism underlying the male-specific association between low SMI and poor OS is unclear, but there are several possible explanations. One is that there is a biological difference in that men typically have more skeletal muscle mass than women, and men lose greater skeletal muscle mass than women as they age [16–18]. Therefore, the correlation between low SMI and frailty is higher in men than in women, and so male GC patients with a low SMI may have poor prognosis. Another reason could be related to the gender difference observed in sarcopenia pathogenesis. Previous studies demonstrated that in men, sarcopenia is induced by an increase in the catabolic hormone myostatin [19, 20]. In contrast, there are no significant differences in myostatin levels between sarcopenic and non-sarcopenic female patients. Nishikawa et al. reported that high myostatin levels are associated with poor OS in patients with benign disease [21], and Kim et al. found that myostatin is an independent predictor of poor OS in patients with metastatic solid cancer [22]. The male-specific relationship between low SMI and poor OS in the current study may reflect the influence of myostatin. This study is retrospective and we don’t have data of myostatin level preoperatively, and so we unfortunately can’t confirm our hypothesis. Validation of impact of myostatin for survival based on gender is needed in a future.

The findings of our study may be useful for identifying patients who would benefit from preoperative plans to increase muscle mass. Resistance exercise and nutritional supplements, even in elderly patients, can increase skeletal muscle mass, lean body mass, and muscle strength and improve walking and physical function [23–25]. Emmelot-Vonk et al. reported that testosterone supplementation in older men significantly increased lean mass compared to the placebo in a randomized clinical study [26]. Interventions that increase muscle mass before surgery or attenuate the reduction in muscle after surgery may be promising treatment approaches that could be effective in improving the prognosis of male GC patients. In particular, Stage 1 GC generally carries no immediate threat, leaving the male patient time to bolster his preoperative nutritional status. Confirmation of the viability of this therapeutic strategy awaits a prospective randomized trial.

The advantage of CT imaging in quantifying skeletal muscle to determine sarcopenia is that preoperative CT examinations are routinely conducted as part of the protocol to clinically stage GC. A recent study found an excellent correlation between the results obtained from dual-energy X ray absorptiometry (DXA) and the analysis of CT images [27]. Thus, we recommend body composition analysis using CT scans for cancer patients. However, wide variability exists in the cut-off values for muscle mass that defines sarcopenia across previously performed studies [28]. We performed reverification of our results using the cut-off value of a first quartile of male and female SMI distribution of this cohort. Nevertheless, a low SMI was still an independent predictor of OS only in male GC patients (data not shown). Nishigori et al. examined which cut-off value was optimal in a cohort of patients with stage 2 or 3 GC using five cut-off values reported previously and concluded that the cut-off value used by Martin et al. was the best for predicting patient outcome (i.e., 53.0 for BMI ≥25 43.0 for BMI <25 in males and 41.0 in females) [3, 29]. However, their study did not include a large number of patients or patients with stage 1 GC. Elderly patients with sarcopenia are not only at risk of death from GC but also other diseases. Therefore, studies that include stage 1 GC are needed. Future work should focus on establishing a definition for sarcopenia using CT measurements to obtain optimal cut-off values for GC patients.

There are several limitations to this study. First, our study was based on data from a single institution. There were also some significant differences in the patient characteristics between men and women. Thus, bias may affect the determined outcomes, and no definitive conclusions can be drawn. Confirmation of our results should be done in multiple facilities. Second, in this study, we evaluated SMI only using CT image. Validation of the relationship between SMI and OS using other devices such as X-ray or bioelectrical impedance is needed. Third, postoperative chemotherapy was not considered in the present study. The low-SMI patients with advanced-stage disease may have received chemotherapy at less than the standard dose. Finally, it is not clear why the impact of preoperative muscle mass on long-term outcomes of gastrectomy differs between male and female patients. Regardless, these findings of this study have a clinical value in helping us to know who we should guide to gain muscle preoperatively to improve survival.

## Conclusions

Assessing muscle mass preoperatively is useful for predicting the prognosis of male GC patients. We believe that interventions that maintain or add to muscle mass before surgery can be more effective in improving the survival of male rather than female GC patients. Preoperative SMI and complications were not related in this setting regardless of gender.

## Supporting information

S1 Table Characteristics of gastric cancer patients. (XLSX)
